# Correction: Arkyurek et al. Gallic Acid, 3-Hydroxytyrosol, and Quercetin Modulate Cholinesterase Activity in *Drosophila melanogaster*. *Int. J. Mol. Sci.* 2026, *27*, 859

**DOI:** 10.3390/ijms27031377

**Published:** 2026-01-30

**Authors:** Tugba Ucar Akyurek, Fatma Sezer Senol Deniz, Ilkay Erdogan Orhan, Memet Gozuboyuk, Gulnur Ipek Erdemli, Guzin Emecen

**Affiliations:** 1 Department of Pharmacognosy, Faculty of Pharmacy, Gazi University, 06330 Ankara, Türkiye; tugba.ucar@tarimorman.gov.tr (T.U.A.); fssenol@gazi.edu.tr (F.S.S.D.); 2 Inovabella Biotechnology and R&D Industry and Trade Limited Company, Health and Drug Technology Centre (LHUSTEK), Lokman Hekim University, 06510 Ankara, Türkiye; 3 Department of Pharmacognosy, Faculty of Pharmacy, Lokman Hekim University, 06510 Ankara, Türkiye; 4 Turkish Academy of Sciences (TÜBA), Vedat Dalokay Cad., No. 112, 06670 Ankara, Türkiye; 5 Functional and Evolutionary Genetics Laboratory (FEGL), Department of Biology, Faculty of Science, Hacettepe University, 06800 Ankara, Türkiye; memetgozuboyuk@hacettepe.edu.tr (M.G.); gulnur.ipek11@hacettepe.edu.tr (G.I.E.); guzin@hacettepe.edu.tr (G.E.)

In the original publication [1], due to the author’s negligence, there was no IRBS and ICS included. The authors state that the scientific conclusions are unaffected. This correction is approved by the Academic Editor. The original publication has also been updated. The new IRBS and ICS should be as shown below:
